# Acute toxicity outcomes in Egyptian early-stage breast cancer: ultra-hypofractionated versus hypofractionated radiotherapy

**DOI:** 10.1186/s43046-025-00280-4

**Published:** 2025-05-03

**Authors:** Ahmed Magdy, Emad Sadaka, Rasha Abd el Ghani, Taha Ahmed

**Affiliations:** 1 Kafrelsheikh University, Kafralsheikh, Egypt; 2 Tanta University, Tanta, Egypt

**Keywords:** Erythema, Desquamation, Acute breast pain, Ultra hypofractionated Radiotherapy, Hypofractionation

## Abstract

**Background:**

Comparison of acute adverse events (acute skin reaction, acute breast pain and lung toxicities) in early-stage breast cancer using 2 different fractionation schedules: ultra-hypofractionation versus hypofractionation radiotherapy.

**Methods:**

Ninety-two patients were recruited and assessed using RTOG criteria for acute skin reactions at the end of radiotherapy, 1 month after, and 3 months after.

**Results:**

There have been no statistically significant differences in acute skin adverse events in 1 month after WBI, there have been neither G3 acute skin toxicity nor G2 skin reactions as were in the fast trial, and milder than skin adverse events in the FAST-FORWARD trial. Acute breast pain at the end of radiotherapy has been statistically significantly lower in arm 1 vs arm 2. Acute breast pain at 1-month follow-up has been comparable between the study arms, with no statistically significant difference. At the 3-month follow-up, acute breast pain was similar in both arms. In all arms, no acute lung toxicities have been reported.

**Conclusion:**

Acute adverse events have been comparable between ultra-hypofractionation and hypofractionation.

## Background

Breast neoplasm is recognized as the most prevalent cancer among females, with case numbers steadily increasing across both developed and developing nations [1]. The implementation of screening programs that facilitate early detection, along with advancements in therapeutic interventions, has significantly enhanced survival rates for individuals diagnosed with breast cancer. Comprehensive management of breast cancer is typically tailored to the specific stage of the disease and may include a combination of surgical intervention, pharmacological treatments, and radiation therapy. In the postoperative care of breast cancer, whether following a conservative approach [2, 3] or radical mastectomy, the commonly adopted radiation fractionation schedule consists of delivering a total of 45–50 Gy through 25 fractions, with each fraction administering 1.8 to 2 Gy per day, five days per week [4, 5].

Elderly patients frequently present with multiple concurrent medical conditions, cognitive impairments, and social limitations [6]. These factors often complicate their ability to travel long distances to healthcare facilities, making it challenging for them to attend daily treatment sessions over extended periods. To enhance accessibility and adherence to treatment, it is advantageous to develop shorter treatment protocols for elderly patients that maintain comparable tumor control outcomes [7].

Hypofractionated radiotherapy delivers a higher dose per fraction, thereby reducing the overall duration and associated costs of the treatment course. Research indicates that postoperative hypofractionated radiation protocols achieve similar locoregional tumor control and have comparable side effects to traditional treatment protocols. As a result, hypofractionation has increasingly become the preferred method for managing treatment in many patients [8–10].

The FAST-Forward trial was conducted with a cohort of 4,096 patients diagnosed with invasive breast carcinoma and aimed to evaluate three distinct schedules of radiation fractionation. These included hypofractionation, comprising 40 Gy delivered in 15 fractions over three weeks, and two forms of ultra-hypofractionated radiotherapy, specifically 27 Gy and 26 Gy, each administered in five fractions. Most participants demonstrated a lower risk profile, with an average age exceeding 50 years and a diagnosis of grade 1 or 2 breast cancer. Findings from the trial revealed that the 27 Gy regimen was associated with significantly higher clinician-assessed normal tissue effects in the breast or chest wall compared to the 40 Gy group, with a p-value of less than 0.0001 at the five-year mark. Conversely, the 26 Gy treatment yielded outcomes comparable to the 40 Gy arm, indicated by a p-value of 0.20 [10].

Additionally, this study assessed the incidence of acute skin reactions and breast pain among early breast cancer patients receiving either ultra-hypofractionated or hypofractionated radiotherapy.

## Patients and methods

### Ethical review statement

This study was approved by the Institutional Review Board (IRB), and all patients signed informed consent.

### Study design

Prospective study to compare ultra hypofractionated radiotherapy versus hypofractionation in terms of acute radiation adverse events according to RTOG criteria.

### Description of the selection of the observational subjects

Ninety-two patients with early-stage breast neoplasia treated between November 2021 and January 2022 have been included.

### Study setting

Randomized prospective study using permuted blocks randomization to 2 nearly equal comparable arms.

### Study duration

Twenty-four months follow up.

### Sampling method

Based on a previous study by Agrawal et al. [11] and Brunt et al. [12], to compare the difference in the acute skin toxicity rate between the 2 arms. The margin for non-inferiority was set to be δ = 1.5%. Given that the true mean acute toxicity rates of the different arms are θ1 = 1.9% and θ3 = 13.6%, respectively. To get an 80% power (β = 0.2) at α = 0.1, the needed sample size with equal allocation (*r* = 1) can be determined by n1 = n2 = 36. The number has increased to a total sample size of 92 to allow for losses of around 25%. The sample size was calculated using Sealed Envelope Ltd. 2012.

### Sample size calculations

Data has been analyzed using IBM SPSS advanced statistics, version 23 (SPSS Inc., Chicago, IL). The estimation of the frequency of acute toxicity grades according to the RTOG toxicity assessment has been described as percentages and numbers.

### Follow-up period

All patients have been examined after treatment sessions for tolerance and received early management of acute radiation adverse events according to the RTOG scale, including, when needed, supportive treatment. Patients were followed regularly at the end of treatment, after a month, and then 3 months.

RTOG Acute radiation morbidity scoring criteria:

- G0: No change from baseline.
- G1: Follicular, faint, dull erythema/epilation/dry desquamation/decreased sweating.
- G2: Tender or bright erythema, patchy moist desquamation/moderate edema.
- G3: Confluent, moist desquamation other than skin folds, pitting edema.
- G4: Ulceration, hemorrhage, necrosis.

### Inclusion and exclusion criteria

The inclusion criteria for the study are as follows: participants must be over 18 years of age and possess good performance status (ECOG ≤ 2). They should have a pathologically confirmed diagnosis of breast cancer with negative surgical margins (pT1–2, pN0, M0) and must have undergone breast-conserving surgery. Furthermore, adequate axillary staging and/or dissection or sentinel lymph node biopsy (SLNB) is required. Postoperative systemic therapy will be provided based on the recommendation of the patient's clinical oncologist. Patients with positive hormone receptors are to receive endocrine therapy for a minimum of five years. Additionally, HER2-positive patients will be administered Trastuzumab. A comprehensive overview of patient characteristics is presented in Table 1.

**Table 1:** The characteristics of the patients

Characteristics All patients	Arm 1			Arm 2				Total		*P* value
	**N**		**(%)**	**N**		**(%)**		**N**	**(%)**	
	**45**		**(48.91%)**	**47**		**(51.09%)**		92	**(100%)**	
**Patients Characteristics**										
**Age (years)**										
≤ 40 years	7		15.6	6		12.8	13		14.13	**0.8619**
41–49 years	14		31.1	14		29.8	28		30.43	**0.9010**
≥ 50 years	24		53.3	27		59.4	51		55.44	**0.5575**
**Marital Status**										
married	44		97.77	45		95.74	89		96.74	**0.5861**
Non married	1		2.23	2		4.26	3		3.26	
**Breastfeeding**										
Yes	41		91.11	40		85.11	81		88.04	**0.3778**
No	4		8.89	7		14.89	11		11.96	
**Oral Contraceptive Pills (OCPs)**										
Yes	17		37.78	18		38.3	35		38.04	**0.9593**
No	28		62.22	29		61.7	57		61.96	
**Menopausal status**										
Pre-menopausal	21		46.67	22		46.81	43		46.74	**0.9893**
Post-menopausal	24		53.33	25		53.19	49		53.26	
**Medical conditions and co-morbidities**										
Hypertension	10		22.22	7		14.9	17		18.48	**0.3685**
Cardiac disease	1		2.22	2		4.26	3		3.26	**0.5840**
Diabetes Miletus	11		24.44	12		25.53	23		25	**0.9045**
**Tumor Characteristics**										
**Tumor side**										
Right sided	27		60	40		85.11	67		72.83	**0.0071**
Left sided	18		40	7		14.89	25		27.17	
**Tumor location**										
Upper Outer quadrant (UOQ)	29		64.44	27		57.45	56		60.87	**0.4946**
Upper inner quadrant (UIQ)	7		15.56	5		10.64	12		13.04	**0.4861**
Lower Outer Quadrant (LOQ)	1		2.22	3		6.38	4		4.35	**0.3305**
Lower Inner Quadrant (LOQ)	2		4.44	7		14.89	9		9.87	**0.0934**
Central	6		13.33	5		10.64	11		11.96	**0.6926**
**Histopathology**										
IDC	44		97.77	47		100	91		98.91	**0.3059**
ILC	1		2.23	0		0.0	1		1.09	
**Grade**										
Grade I	2		4.44	0		0.0	2		2.17	**0.5081**
Grade II	31		68.88	34		72.34	65		70.65	**0.7171**
Grade III	12		26.68	13		27.66	25		27.18	**0.7171**
**T stage**										
T1mic	0		0.0	1		2.13	1		1.09	**0.3276**
T1b	1		2.22	2		4.26	3		3.26	**0.5840**
T1c	21		46.66	14		29.79	35		38.03	**0.0975**
T2	23		51.12	30		63.82	53		57.62	**0.2204**
**Hormone receptors and Her2 status**										
Positive hormone receptors	41		91.11	40		85.10	81		88.04	**0.3771**
Her2 enriched	8		17.78	11		23.4	19		20.65	
**Chemotherapy**										
No	18		40	9		19.15	27		29.35	**0.0277**
Yes	27		60	38		80.85	65		70.65	
**Treatment policy**										
WBI	14		31.11	18		38.3	32		34.78	**0.4716**
WBI + boost	31		68.89	29		61.7	60		65.22	

*N* Number of cases, *IDC* Invasive ductal carcinoma, *ILC* Invasive lobular carcinoma, *AJCC* American Joint Committee on Cancer, *WBI* Whole breast irradiation

The following criteria outline the exclusions for the study:

- Patients diagnosed with Tis (ductal carcinoma in situ)
- Patients classified as pT3-4 or pN + with breast cancer
- Male patients
- Patients who have undergone modified radical mastectomy
- Individuals with musculoskeletal deformities that impede their ability to lie in the required treatment position
- Patients with inadequate axillary lymph node dissection, defined as the removal of fewer than 10 lymph nodes
- Patients with pathologically confirmed positive surgical margins following breast-conserving surgery
- Individuals diagnosed with triple-negative breast cancer (TNBC)
- Patients who have received neoadjuvant chemotherapy
- Individuals with non-epithelial breast malignancies, including sarcoma or lymphoma
- Patients presenting with synchronous bilateral invasive or non-invasive breast cancer
- Individuals with a history of invasive breast cancer or ductal carcinoma in situ (DCIS)
- The presence of cosmetic breast implants
- Patients who have previously received breast or thoracic radiation therapy for any condition
- Individuals with collagen vascular diseases, particularly systemic lupus erythematosus or scleroderma
- Those who are pregnant or lactating at the time of radiation therapy
- Patients with serious, uncontrolled physical or cerebrovascular disorders (e.g., myocardial infarction within the last 12 months)
- Individuals with neurological or psychiatric disorders that may negatively impact treatment compliance.

These criteria ensure a focused approach that prioritizes patient safety and treatment efficacy.

### Radiotherapy technique

WBI begins within 9 weeks following conservative surgery if the patient is not indicated for postoperative chemotherapy. However, if chemotherapy is indicated, WBI starts between 2 and 8 weeks after the last chemotherapy session.

A linear accelerator is used to treat all patients with 3D conformal tangential beams targeting the breast volume. Patients are positioned supine with the ipsilateral arm abducted, utilizing an alpha cradle. Thin CT slices, averaging 5mm in thickness, are obtained from the chin to the upper abdomen. All target volumes are contoured in the planning system on these CT slices according to our protocol, following the "RTOG Breast Cancer Atlas."

**Fig. 1:**
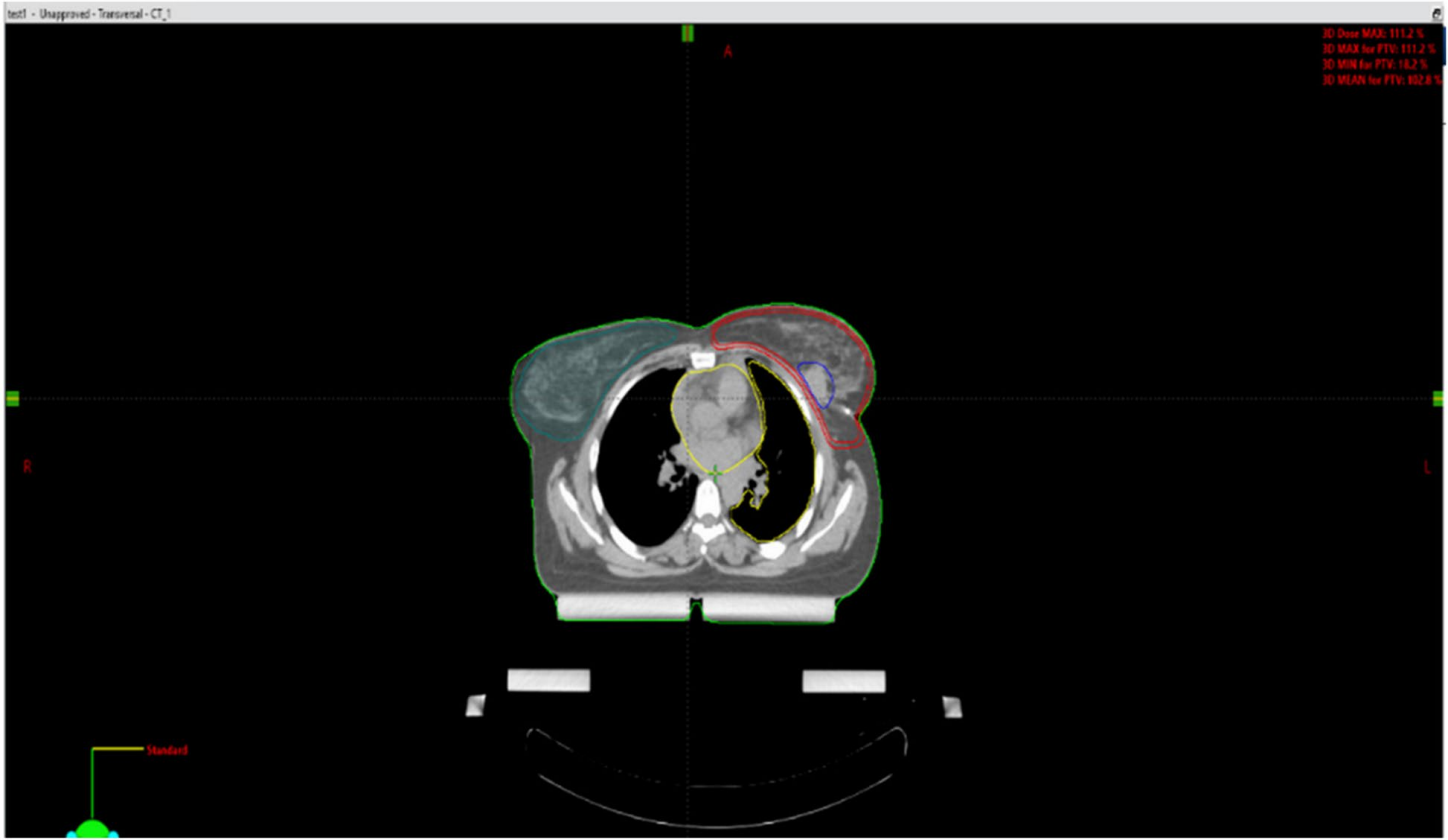
Contouring of target volumes CTV left breast (light red), PTV left breast (Dark red), and boost target volume (dark blue), organs at risk: contralateral breast (Turquoise), heart (yellow), left lung (light green)

**Fig. 2:**
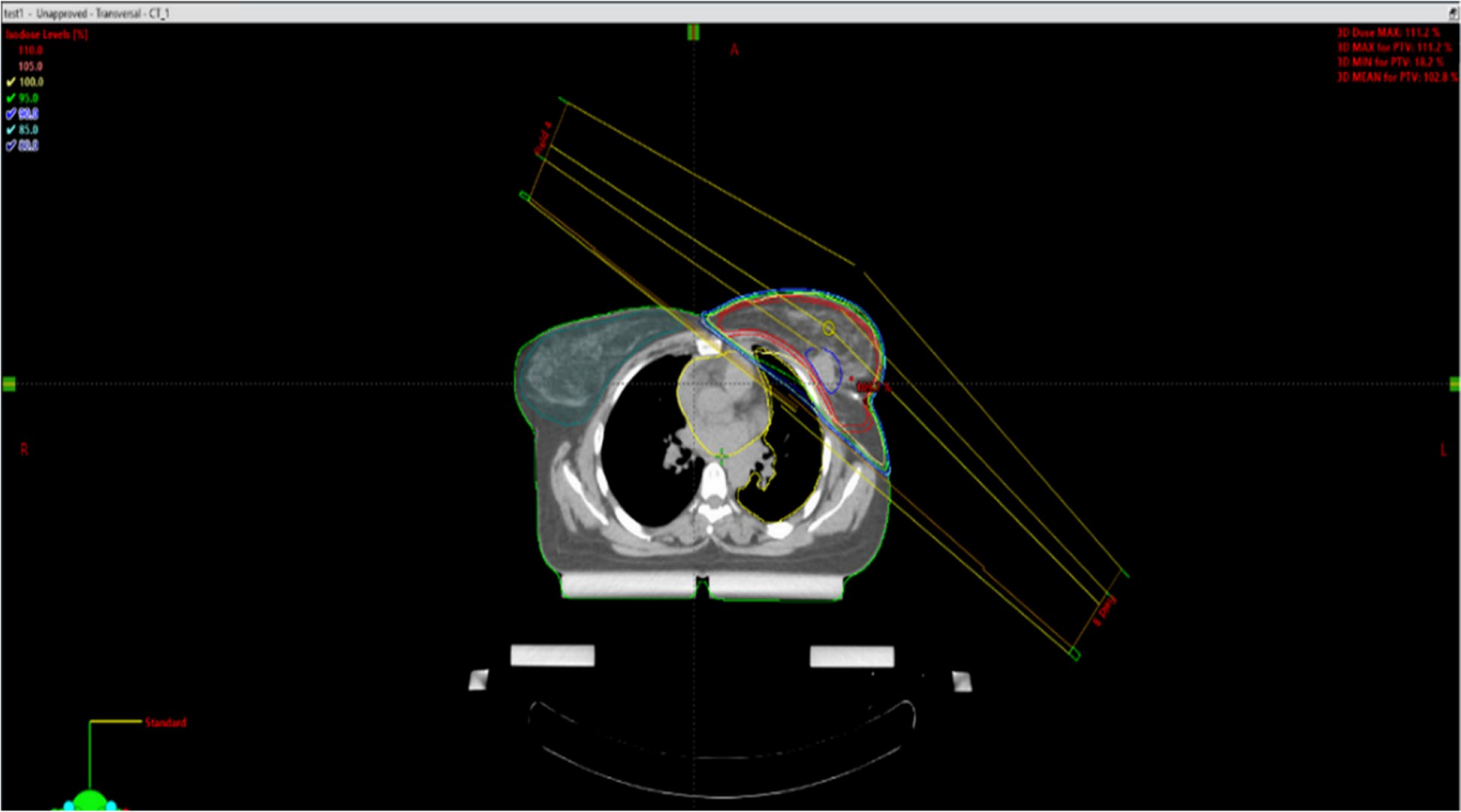
Contouring of PTV left breast (dark red) with field arrangement for the 40.05 Gy arm

An auto-contouring tool is employed to delineate the outer body contour, Breast Clinical Target Volume (CTV), Planning Target Volume (PTV), Lumpectomy Clinical Target Volume (CTV), Lumpectomy Planning Target Volume (PTV), Boost Clinical Target Volume (B-CTV), and Boost Planning Target Volume (B-PTV) [13]. These volumes are contoured based on the anatomical definitions outlined in the RTOG Breast Cancer Atlas, as illustrated in Figs. 1 and 2.

For Lumpectomy Target Volumes, a boost to the tumor bed is highly recommended for higher-risk patients—specifically those under 50 years old, with high-grade disease, or those with focally positive margins, according to NCCN guidelines. The lumpectomy represents the surgical cavity resulting from breast-conserving surgery. Contouring utilizes all available clinical and radiographic information, including seroma and the extent of surgical clips or the lumpectomy scar.

**Fig. 3:**
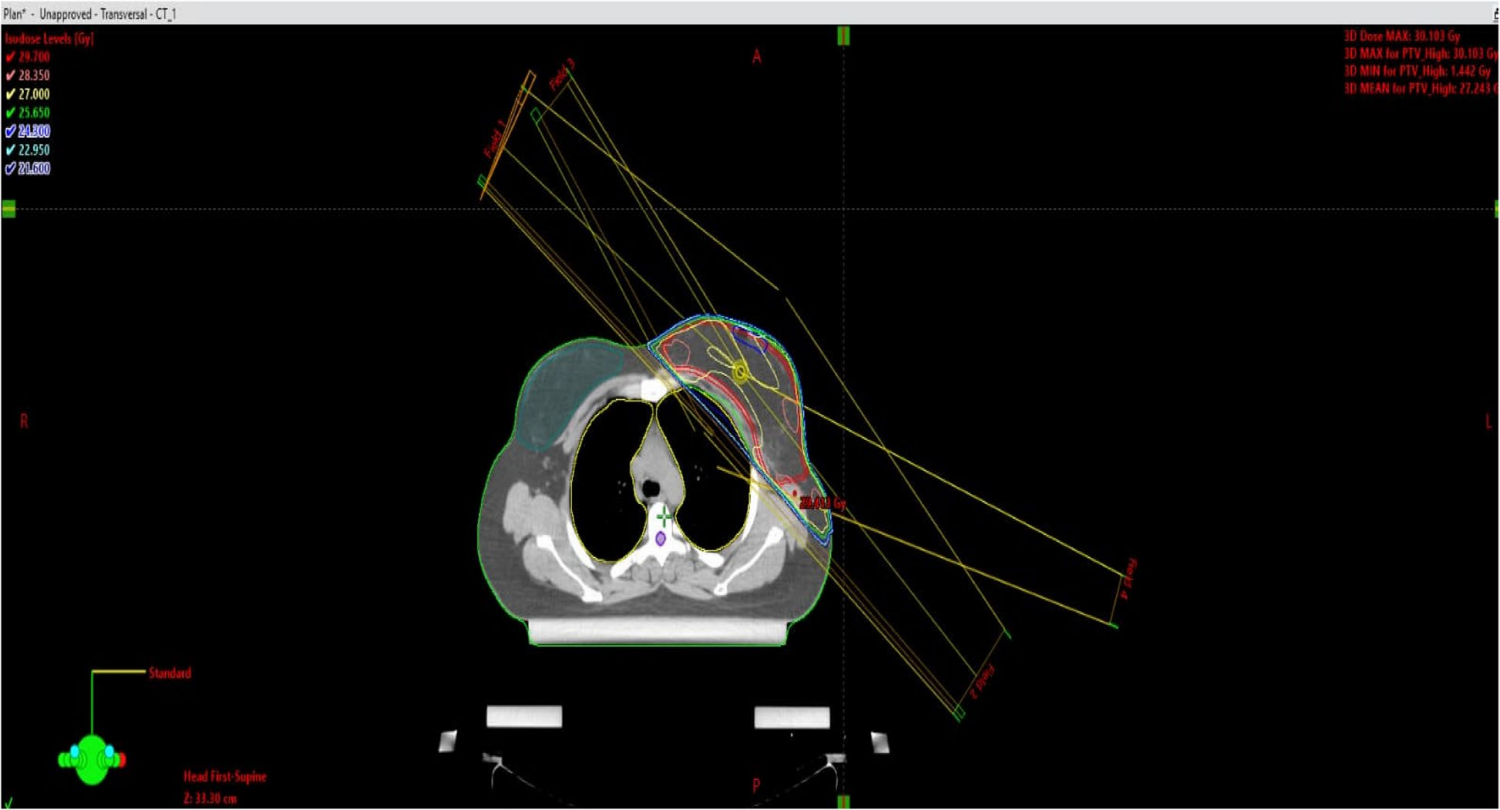
Contouring of target volumes PTV left breast (dark red), with field arrangement for the 27 Gy arm

Additionally, Organs at Risk (OAR) are contoured on all CT cuts and include the heart, spinal cord, contralateral breast, both lungs, and the thyroid gland, as depicted in Fig. 3.

Two radiation schedules were compared:

- 1st arm ultrahypofractionated radiotherapy of 27 Gy in 5fr over 1 week. 5.4Gy/fr ± boost to the tumor bed if indicated of 0.50 Gy per fraction, for a total dose of 29.5 Gy [14].
- 2nd arm hypofractionated radiotherapy in the form of 40.05 Gy/ 15 fractions/ 3 weeks. 2.67 Gy/ fraction ± boost to the tumor bed if indicated of 0.53 Gy per fraction, for a total dose of 48 Gy [15].

The treating physicians have sanctioned the implementation of daily setup verification utilizing electronic portal images (EPI) for all participants involved in the study. This verification process is mandated before initiating the protocol and before each session within the first treatment arm. To address any systematic errors, both EPI and necessary corrections will be conducted during the initial three treatment sessions. Following this period, EPI will be required weekly. A tolerance of 5 mm has been established as acceptable.

## Results

The study involved a total of ninety-two patients. The majority resided a significant distance from the healthcare center, which presented challenges in accessing timely treatment. Each patient underwent breast-conserving surgery, with 75.5% receiving a lumpectomy and 24.5% undergoing a quadrantectomy. An overview of acute radiation-related adverse events is presented in Table 2.

**Table 2:** The RTOG acute radiation adverse events encountered in patients during follow up

Characteristics All patients	Arm 1		Arm 2		Total		*P*-value
	No	(%)	No	(%)	No	(%)	
	45	48.91	47	51.09	92	100	
**RTOG acute skin reactions** **At the end of WBI**							
** G0**	38	84.6	14	30	52	56.52	**< 0.0001**
** G1**	7	15.4	30	64	37	40.22	**< 0.0001**
** G2**	0	0	3	6	6	3.26	**0.0969**
**1 month after WBI**							
** G0**	10	22.22	12	25.53	22	23.91	**0.7113**
** G1**	33	73.33	34	72.34	67	72.83	**0.9155**
** G2**	2	4.45	1	2.13	3	3.26	**0.5336**
**3 months after WBI**							
** G0**	17	37.78	15	31.91	32	34.78	**0.5567**
** G1**	22	48.89	32	68.09	54	58.7	**0.0630**
** G2**	6	13.33	0	0	6	6.52	**0.0100**
**RTOG acute breast pain** **At the end of WBI**							
** G0**	35	77.78	19	40.43	54	58.7	**0.0003**
** G1**	10	22.22	26	55.32	36	39.1	**0.0012**
** G2**	0	0	2	4.25	2	2.2	**0.1644**
**1 month after WBI**							
** G0**	14	31.11	13	27.66	27	29.35	**0.7179**
** G1**	22	48.89	28	59.57	50	54.35	**0.3066**
** G2**	9	20	6	12.77	15	16.3	**0.3507**
**3 months after WBI**							
** G0**	22	48.89	27	57.45	49	53.26	**0.4133**
** G1**	20	44.44	14	29.78	34	36.96	**0.1475**
** G2**	3	6.67	6	12.77	9	6.78	**0.3276**

*N* Number of cases, *G* Grade, *WBI* Whole breast irradiation

### Acute radiation skin reactions

Acute Grade 1 (G1) radiation dermatitis emerged as the most frequently reported adverse event in the study. Mometasone cream was administered twice daily for a duration of 5 days to patients exhibiting skin erythema. Subsequently, a beta-sitosterol cream was applied for two weeks.

#### Arm 1

**Fig. 4:**
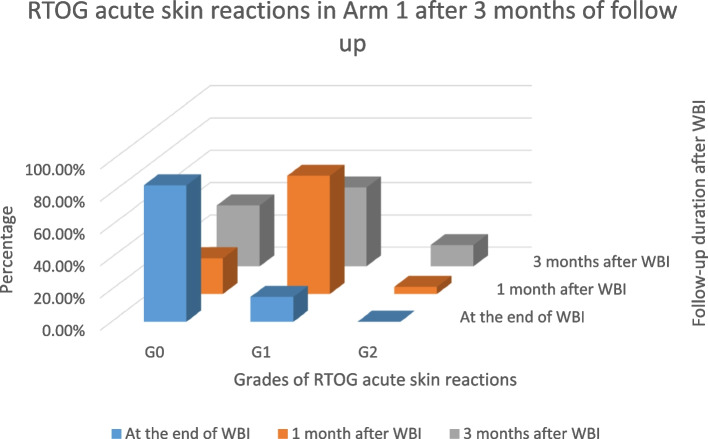
RTOG acute skin reactions in Arm 1 after 3 months of follow-up

At the end of WBI, acute skin reactions were classified as Grade 0 (G0) in 38 patients (84.6%) and Grade 1 (G1) in 7 patients (15.4%). One month post-WBI, the classification of acute skin reactions indicated G0 in 10 patients (22.22%), G1 in 33 patients (73.33%), and Grade 2 (G2) in 2 patients (4.45%). After three months, the distribution of acute skin reactions was G0 in 17 patients (37.78%), G1 in 22 patients (48.89%), and G2 in 6 patients (13.33%), as illustrated in Fig. 4. All cases classified as G2 were characterized by tender, bright erythema without any desquamation.

#### Arm 2

**Fig. 5:**
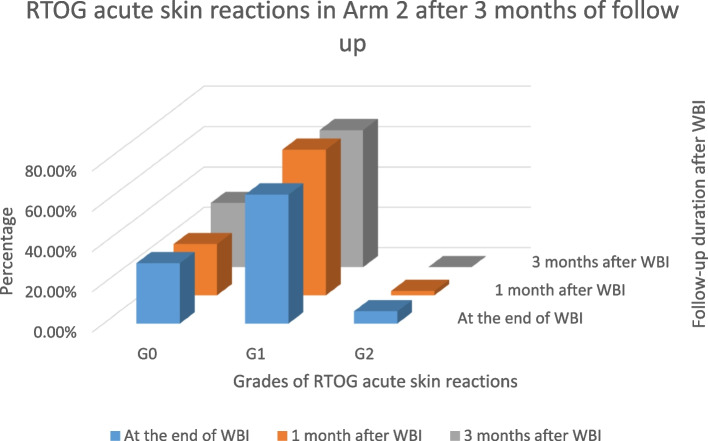
RTOG acute skin reactions in Arm 2 after 3 months of follow-up

By the end of WBI, skin reactions in this arm were classified as G0 in 14 patients (30%), G1 in 30 patients (64%), and G2 in 3 patients (6%). Three patients experienced patchy moist desquamation, which was treated with beta-sitosterol cream, resulting in healing within two weeks. One month following WBI, acute skin reactions were recorded as G0 in 12 patients (25.53%), G1 in 34 patients (72.34%), and G2 in 1 patient (2.13%). After three months, the results were G0 in 15 patients (31.91%) and G1 in 32 patients (68.09%), as depicted in Fig. 5.

A comparative analysis of acute skin reactions between the two treatment arms at the end of WBI revealed a statistically significant difference, with Arm 1 exhibiting a higher incidence of reactions than Arm 2. This disparity was anticipated, as the one-week treatment regimen was likely insufficient to facilitate adequate epidermal repopulation and radiosensitization due to reassortment. One month post-WBI, both arms exhibited comparable acute skin reactions, with no statistically significant differences observed. However, after three months of treatment, a statistically significant increase in acute skin reactions was noted in Arm 1 when compared to Arm 2.

### Acute breast pain

Most patients who presented with acute breast pain were classified as having grade 1 pain, while a smaller proportion experienced grade 2 pain and were effectively managed with non-steroidal anti-inflammatory drugs (NSAIDs).

#### Arm 1

**Fig. 6:**
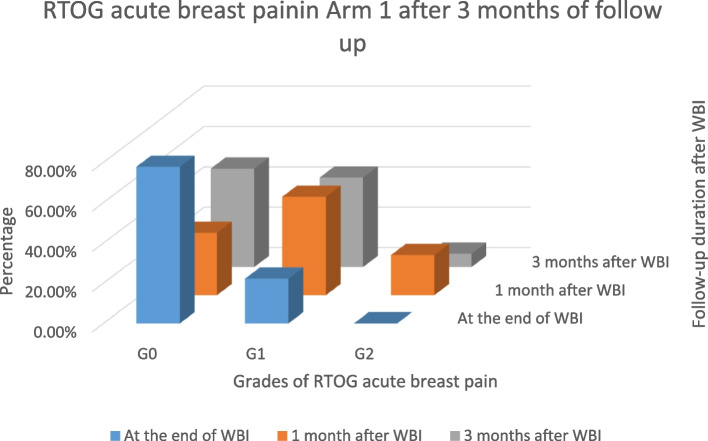
RTOG acute breast pain in Arm 1 after 3 months of follow-up

At the end of WBI, acute breast pain was categorized as grade 0 (G0) in 35 patients (77.78%) and grade 1 (G1) in 10 patients (22.22%). One month following WBI, pain assessment revealed G0 in 14 patients (31.11%), G1 in 22 patients (48.89%), and grade 2 (G2) in 9 patients (20.0%). After three months post-WBI, pain was classified as G0 in 22 patients (48.89%), G1 in 20 patients (44.44%), and grade 3 (G3) in 3 patients (6.67%), as illustrated in Fig. 6.

#### Arm 2

**Fig. 7:**
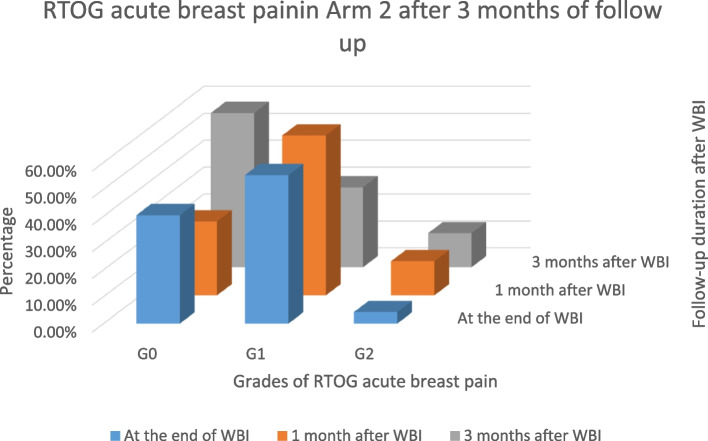
RTOG acute breast pain in Arm 2 after 3 months of follow-up

By the end of WBI, acute breast pain was recorded as G0 in 19 patients (40.43%), G1 in 26 patients (55.32%), and G2 in 2 patients (4.25%). One month post-WBI, the assessment indicated G0 in 13 patients (27.66%), G1 in 28 patients (59.57%), and G2 in 6 patients (12.77%). Following three months of WBI, pain assessment identified G0 in 27 patients (57.45%), G1 in 14 patients (29.78%), and G2 in 6 patients (12.77%), as shown in Fig. 7.

Comparative analysis of acute breast pain between the two arms indicated that pain levels at the end of WBI were statistically significantly lower in Arm 1 than in Arm 2. At the one-month follow-up, acute breast pain levels were comparable between the two study arms, with no statistically significant difference observed. After three months of WBI, pain levels remained similar in both arms. Importantly, no acute lung toxicities were reported in any of the arms involved in the study.

## Discussion

In our study, we carefully evaluated and recorded acute radiation adverse events for all consenting patients. Notably, there were no Grade 3 or higher acute skin adverse events reported in any of the study groups. This positive outcome can be attributed to the preventive measures implemented in the study, which included behavioral practices and the use of topical agents.

Several dosimetric factors influence skin adverse events. A higher total radiation dose, the addition of a surgical bed boost, a dose distribution inhomogeneity exceeding 107% of the prescribed total dose, and increased volumes of hotspots in the tissues are all associated with a greater risk of skin adverse events [16–19]. The fractionation schedules have a significant impact on the occurrence of acute skin adverse events. Various studies indicate that conventionally fractionated radiotherapy (CF-RT) is associated with a higher incidence of moist desquamation, dermatitis, pruritus, and hyperpigmentation when compared to hypo-fractionated breast protocols [20–22].

In the FAST trial, it is important to note that clinical assessments of acute skin adverse events were not available for all recruited patients, as this aspect was integrated into the trial data collection at a later stage. Among patients who received ultrahypofractionated radiotherapy with a 28.5 Gy schedule, G1 skin reactions were observed in 50% of cases, while G2 reactions were present in 8.5%, and G3 reactions were recorded in 1.9% of patients. In our study, we observed no statistically significant differences in acute skin adverse events one month following WBI. Notably, there were no cases of Grade 3 (G3) acute skin toxicity, and the incidence of Grade 2 (G2) skin reactions was comparable to that reported in the FAST and the FAST-Forward trial.

FAST-Forward was the first robust randomized trial involving 4,100 patients that supported using a 5-day radiotherapy schedule for most patients with early breast cancer. This trial compared a one-week course of curative whole-breast radiotherapy with the conventional 3-week regimen, assessing both local tumor control and side effects in early breast cancer cases. Overall, the results indicate that the 1-week course achieves comparable local control and toxicity profiles to the traditional 3-week treatment. Although this high-quality study has paved the way for this approach, additional randomized and observational studies are required to either confirm or refute its findings. The 5-fraction schedule has not yet become a standard practice in many radiotherapy centers, partly due to concerns over the limited 5-year follow-up, with 10-year results still pending. Our study's findings are in line with those of the FAST-Forward trial. This treatment approach has gained relevance during the COVID-19 pandemic, as shorter treatment schedules help reduce potential exposure for both patients and healthcare staff. However, there is a logistic problem: the cost of delivery of radiotherapy depends on the number of fractions, not the quality of the service, and that’s why many centers prefer to use the older conventional or hypofractionation over the ultra-hypofractionation [10].

The early toxicity rates in our study align closely with those documented in similar research. A. Montero et al. reported that during the three months after WBI, Grade 1 (G1) toxicity was observed in 50.4% of patients, while G2 toxicity was noted in 7.8% of cases [23]. Additionally, R. Pathak et al. found that the Radiation Therapy Oncology Group (RTOG) recorded G1 toxicity in 6.6% and G2 toxicity in 0.3% of patients. Furthermore, maximum skin toxicity classified as ≥ G2 at two weeks post-radiation therapy was significantly higher with ultra-hypofractionation compared to the daily regimen [24]. These findings contribute valuable insights into the safety profile of WBI and highlight the differences in skin toxicity associated with varying treatment schedules.

Although the HYPORT‐BC was a single‐arm study, its results have contributed to the overall evidence that a 5‐fraction schedule is safe in terms of acute skin toxicity when compared with historical data from conventional regimens [25].

Pain is the most annoying symptom, affecting about 30% of patients during RT [26] and 50% of patients at the end of RT [27]. Results showed that hypo-fractionation had less acute skin adverse events [28].

In our study, the RTOG scoring system for acute radiation adverse effects has been used to assess acute breast pain. When comparing acute breast pain among the fractionation utilized, acute breast pain at the end of RT was statistically significantly lower in arm 1 vs arm 2 (*p* value 0.0003). After one month of WBI, acute breast pain was similar between the study arms (*p*-value > 0.05). At the 3-month follow-up, acute breast pain was similar for both arms (*p*-value > 0.05).

The Fast trial didn’t investigate Acute breast pain. In the WHBI trial, the rate of ≥ G2 radiation-induced pain was 17.7%. The FAST-Forward trial reported breast pain as a part of PROMs. There were no reported cases of acute radiation-induced lung or heart adverse events in the study arms.

The current study has several limitations. First, the small sample size. Additionally, because this was a single-center study, further research involving multiple institutions and extended follow-up is needed to reduce potential bias. Although the study received Institutional Board approval and was randomized, it was not registered as a clinical trial.

## Conclusion

Patient and clinician-assessed normal tissue effects in breast appearance after ultra-hypofractionation is non-inferior to hypofractionated radiotherapy.

## Data Availability

The data that support the findings of this study are available from the corresponding author, upon reasonable request. The authors declare no competing interests.
